# Dissociating the effect of disruptive colouration on localisation and identification of camouflaged targets

**DOI:** 10.1038/s41598-018-25014-6

**Published:** 2018-04-26

**Authors:** Rebecca J. Sharman, Stephen J. Moncrieff, P. George Lovell

**Affiliations:** 1 Sharman, Psychology Division, Faculty of Natural Sciences, University of Stirling, Stirling, FK9 4LA UK; 2 Moncrieff & Lovell, School of Social and Health Science, Abertay University, 1, Bell Street, Dundee, DD1 1HG UK

## Abstract

Disruptive camouflage features contrasting areas of pigmentation across the animals’ surface that form false edges which disguise the shape of the body and impede detection. In many taxa these false edges feature local contrast enhancement or edge enhancement, light areas have lighter edges and dark areas have darker edges. This additional quality is often overlooked in existing research. Here we ask whether disruptive camouflage can have benefits above and beyond concealing location. Using a novel paradigm, we dissociate the time courses of localisation and identification of a target in a single experiment. We measured the display times required for a stimulus to be located or identified (the critical duration). Targets featured either uniform, disruptive or edge enhanced disruptive colouration. Critical durations were longer for identifying targets with edge enhanced disruptive colouration camouflage even when presented against a contrasting background, such that all target types were located equally quickly. For the first time, we establish empirically that disruptive camouflage not only conceals location, but also disguises identity. This shows that this form of camouflage can be useful even when animals are not hidden. Our findings offer insights into how edge enhanced disruptive colouration undermines visual perception by disrupting object recognition.

## Introduction

**Figure 1:**
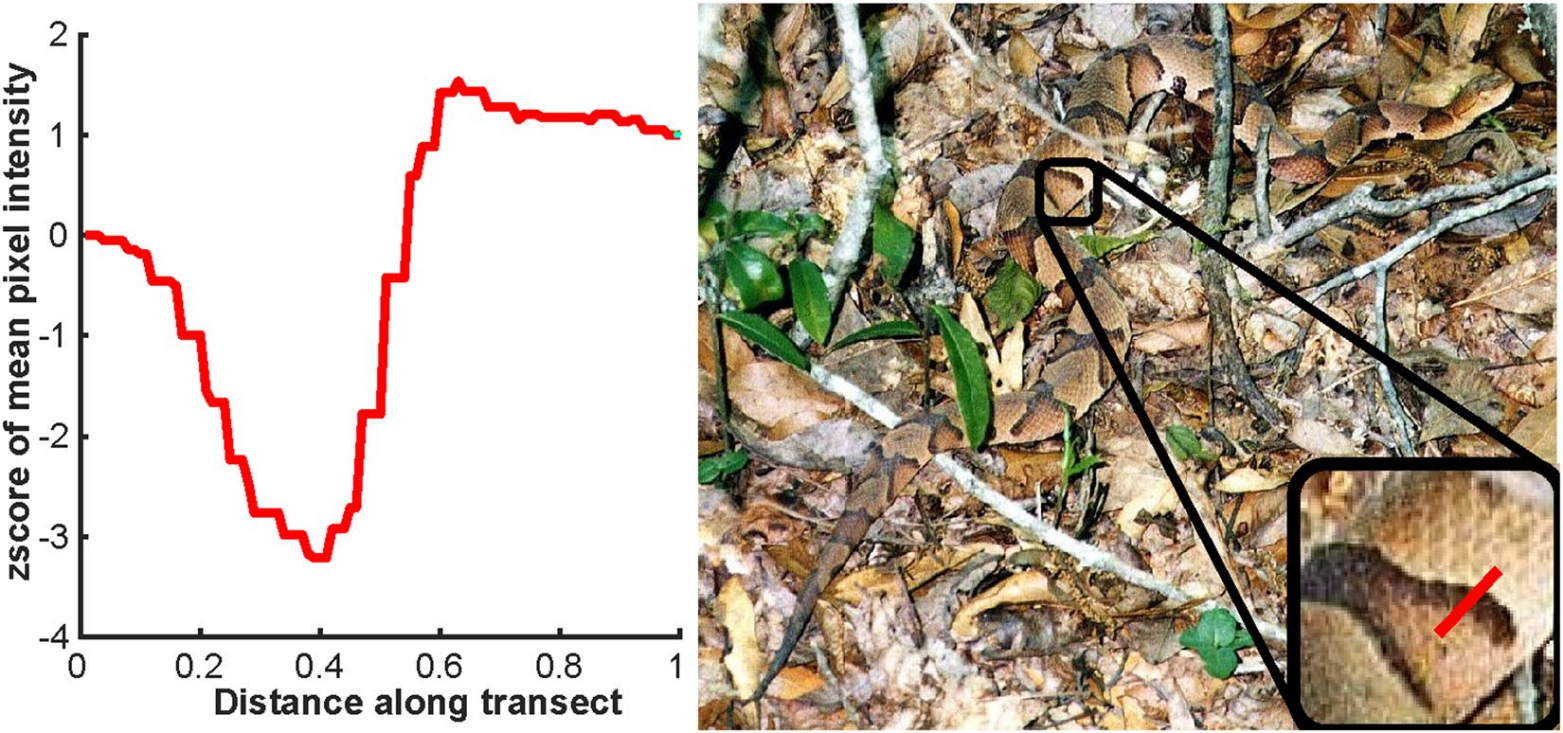
Edge-enhanced camouflage in the copperhead snake (*Agkistrodon contortrix contortrix*). The red line indicates a transect location of the data graphically presented in the left hand plot. NB. The data and image shown were not calibrated to reflect the visual response of any particular organism, we have linearized the intensity values within the images by raising to the power 2.2^21^ and then we calculate the average of red, green and blue pixels within a seven pixel diameter along the transect. Images: (Snake Source wikimedia commons, Tim Ross).

Disruptive colouration is a type of camouflage that consists of contrasting patches of colour which form false edges, disguising the shape of the body and impeding detection^1–3^. Many species with disruptive colouration feature enhanced edges, where light patches have a lighter outline and/or dark patches have a darker outline (see Egan *et al*.^4^, Fig. 1 for an illustration and Fig. 1 below). For example, the lime hawkmoth *Mimas tilae*, the copperhead snake *Agkistrodon contortrix*; the spotted marsh frog *Limnodynastes tasmaniensis*; the leopard *Panthera pardus pardus;* the African hunting dog *Lycaon pictus*; the ball python *Python regius*.

Egan, *et al*.^4^ demonstrated that edge enhanced disruptive camouflage (Fig. 1a) not only improves crypsis, but is also judged by observers to contain more depth; suggesting it improves concealment by giving the impression of pictorial depth across the animal’s surface, mimicking depth variations within background texture^4^. It may also operate by amplifying the effect of the false edges and improving the concealment of the true outline. Conventionally, disruptive camouflage is thought to be concerned primarily with concealment, though it has been speculated that it may impair the cognitive processes underlying recognition^5,6^. Here we ask firstly, whether disruptive colouration can have benefits beyond concealment; is identification slowed for disruptively coloured targets and secondly whether edge enhancement can increase effectiveness in this task? The role of disruptive colouration in disguising the identity of a target has received little empirical attention. Identification of a target has often been conflated with localisation of that target. There has been an assumption that if an observer knows where a target is, they must also know what it is^7^. The key benefit of the current study compared to Egan *et al*.^4^ is the dissociation between localisation and identification. This is an important advance and will demonstrate that disruptive camouflage has benefits above and beyond concealment, showing that we must consider whether targets can be identified as well as located when evaluating the effectiveness of camouflage.

Masquerade provides a useful example of colouration shown to promote misidentification as a different object, even when the target animal is clearly visible^8^. In disruptive colouration identification may be slowed by interfering with recognition processes rather than by misdirecting those processes towards a specific different object. In the current work we use a novel paradigm to dissociate the time courses of localisation and identification in a single experiment. We ask human participants to not only state where a target is, but also identify it as a predator or prey animal. We measure the critical duration; how long a stimulus needs to be presented before judgements become reliably correct. The critical duration is a measure of stimulus presentation time and should not be confused with reaction time.

**Figure 2:**
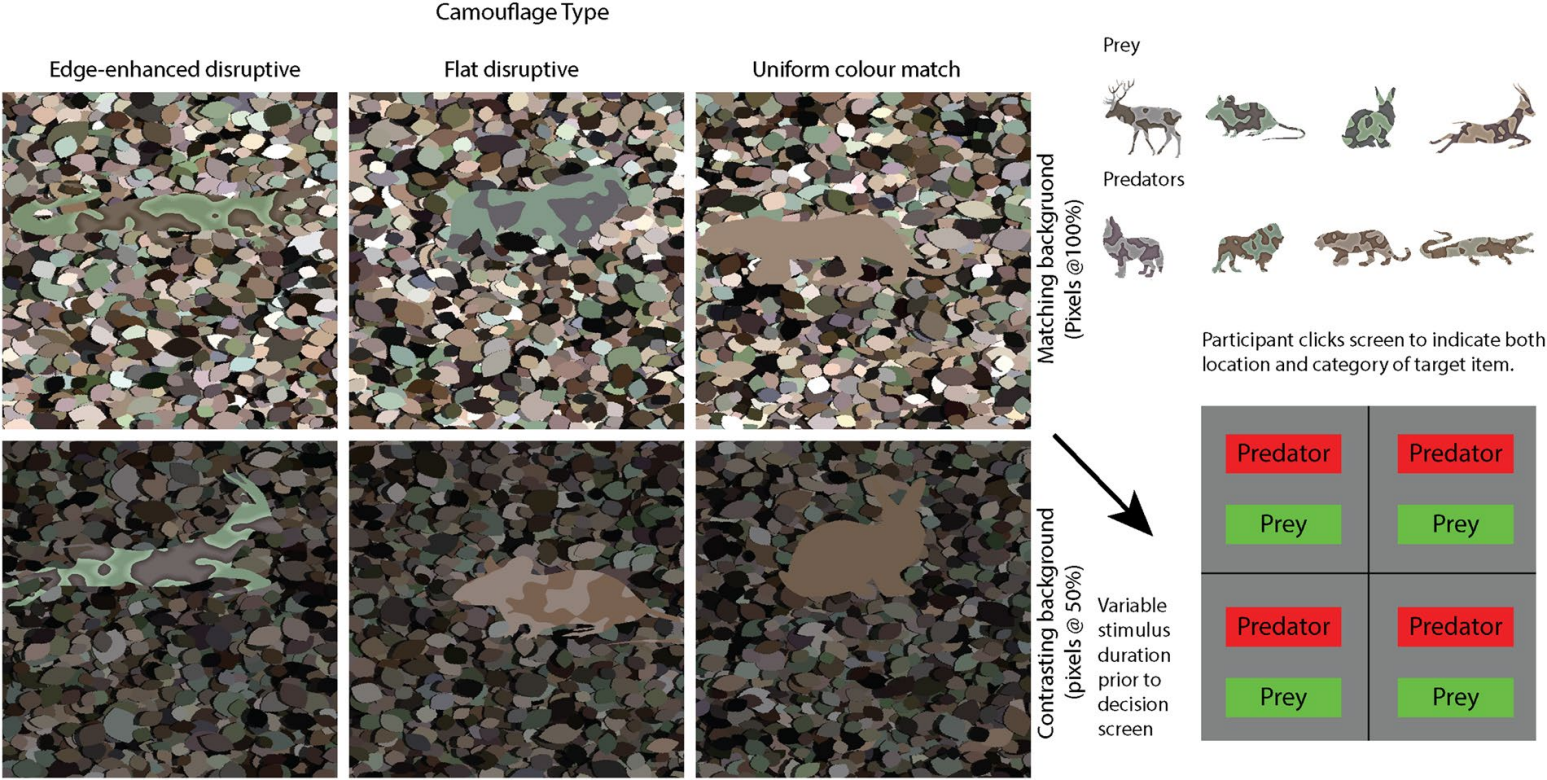
Example stimuli and response image. The left-hand panel of six images shows one quadrant (containing a target) representative of each experimental condition, i.e. the stimulus is 2x larger overall and the target is located in one of the four quadrants. Upper-right, the eight animal shapes used during the experiment. Lower-right, the response image, participants clicked one of the options to indicate category and location. All stimuli were created by the authors in Matlab (Version 8.4, The MathWorks Inc., Natick, MA, 2014) and saved as portable network graphics files.

Targets had three possible types of colouration (edge enhanced disruptive, flat disruptive and uniform) and were presented on two types of background a *matching background* against which targets were relatively concealed (full contrast) and a *contrasting background* against which targets were detected relatively quickly (reduced contrast - where all pixel intensities were halved) as shown in Fig. 2. We predict that:

1. In the matching background condition localisation and identification critical durations will be longer for flat disruptive colouration compared to the uniform control and longer still for edge enhanced disruptive colouration; edge enhancement will increase the effectiveness of disruptive colouration.
2. In the contrasting background condition, localisation critical durations will be comparable for all conditions.
3. In the contrasting background condition identification critical durations will be longer for flat disruptive colouration compared to the uniform control and longer still for edge enhanced disruptive colouration.

## Results

**Figure 3:**
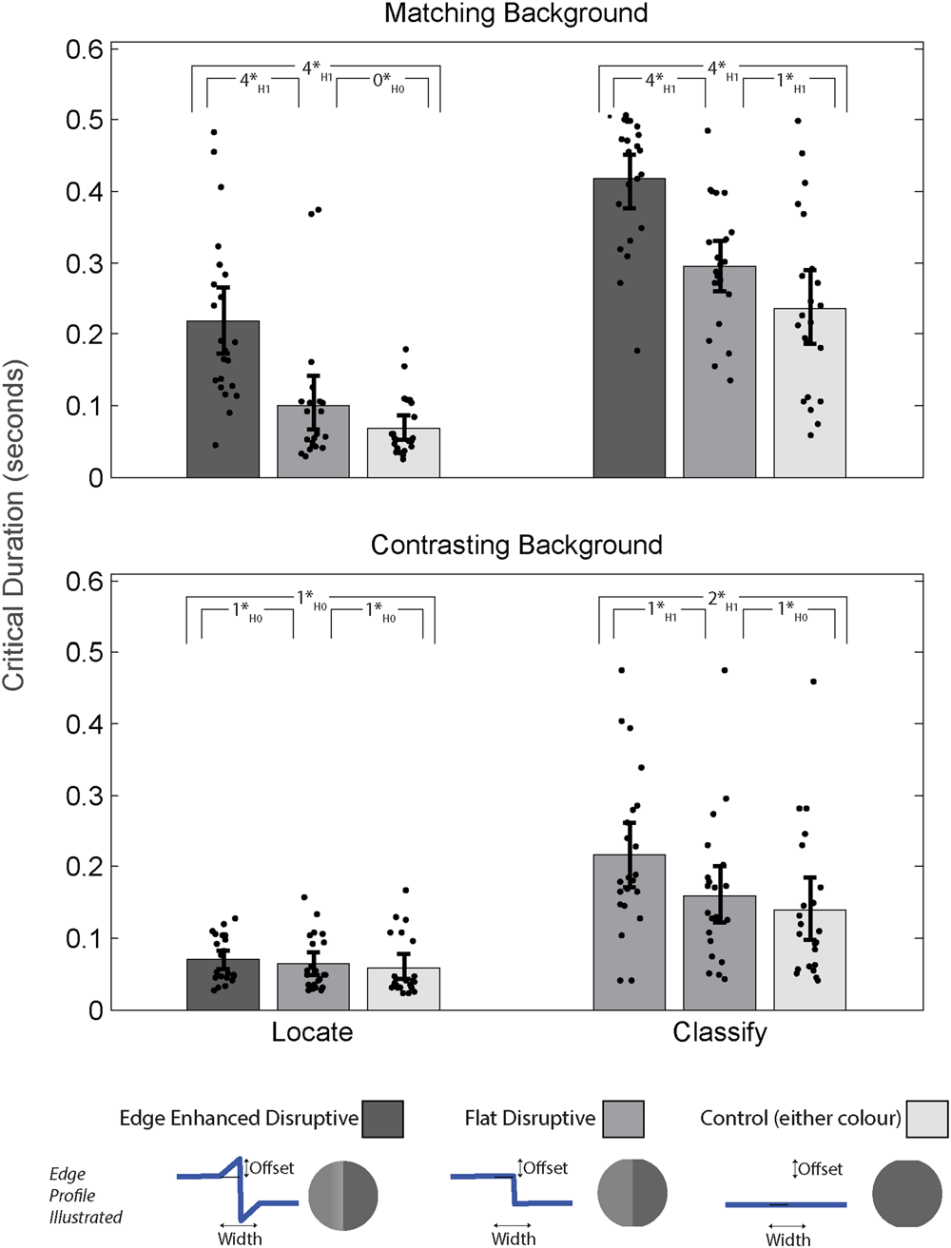
Experiment results. Upper panel shows the results for the full contrast background conditions and the lower panel shows the results for the reduced contrast background conditions. The offset is the change in brightness (CIE lum units) relative to the neighbouring coloured patch. The width is the distance over which the edge enhancement varies, measured from the boundary between the two contrasting patches. All error bars represent the bootstrapped 95% confidence intervals for the reaction times; dots are jittered raw data. Inset at the top of each plot is a summary of the Bayesian t-test results: 0* = anecdotal, 1* = moderate, 2* = strong, 3* = very strong and 4* = extreme evidence for H_0_ or H_1_ (the null and experimental hypotheses respectively).

The raw data analysed in this section are available for download from the Open Science Framework^9^. Figure 3 shows the averaged critical duration for each experimental condition. Prior to statistical analysis we z-score-transformed the 12 thresholds measured for each participant, eliminating some sources of inter- and intra-participant variability. With a matching stimulus background, the edge enhanced colouration targets had a longer critical duration for localisation (>0.2 s) than targets with flat disruptive colouration (<0.1 s) and those with uniform colouration (≈0.07 s). With a contrasting stimulus background there was no difference between any of the colourations. Targets with flat and edge enhanced disruptive colouration had longer critical durations for identification when presented against a matching background. When targets were presented against a contrasting background only the edge enhanced colouration had a longer critical duration for identification.

We use a Bayesian approach to data analysis because it offers robust and transparent inferences. Bayesian analyses compare the odds for both the null and experimental hypothesis, thereby reducing the likelihood of a Type-I error^10^. However, it should be noted that our key results remain statistically significant if analysed with frequentist statistics and have large effect sizes. For full details on this tests please refer to the Open Science Framework^9^.

We conducted a Bayesian analysis of variance (ANOVA) on the critical durations for localisation and identification (JASP^11^; Table S1). The preferred model with the largest Bayes factor (BF10 = 6.075 × 10^61^), included all 2-way interactions (between colouration type, background type, and task type), but did not include the 3-way interaction between all terms. In order to unpack what these differences mean we conducted Bayesian within-subjects t-test analyses (JASP^11^; Table S2), criteria were based on Jeffreys^12^ and Lee and Wagenmakers^13^.

For the localisation critical durations on matching backgrounds there was extreme evidence that edge enhanced disruptive colouration required a longer localisation critical duration than uniform colouration (BF = 94715) and flat disruptive colouration (BF = 589) giving odds-ratios of greater than 100:1 in favour of the experimental hypotheses. Critical durations for flat disruptive colourations were not different from those for uniform colouration (BF = 0.838, odds ratio ≈ 1:1). For the contrasting background conditions localisation critical durations were comparable for all three colourations (moderate evidence, Bayes Factors ≈ 0.25, or 1:4 in favour of the Null Hypothesis).

For the identification critical durations on matching backgrounds there was extreme evidence that edge enhanced disruptive colouration required a longer critical duration than either flat disruptive colouration (BF = 1674, odds ratio = 1674:1) or uniform colouration (BF = 2389, odds ratio = 2389:1). There is also moderate evidence that flat disruptive colouration required a longer identification critical duration than uniform colouration (BF = 3.7 or 4:1).

For the contrasting background conditions identification critical durations are longer for edge enhanced colouration compared to uniform colouration (strong evidence, BF = 13.23, odds ratio = 13:1) and flat disruptive colouration (moderate evidence, BF = 4.75, odds ratio = 5:1). There was moderate support for there being no difference between uniform colouration and flat disruptive colouration (BF = 0.302, odds ratio 3.3:1 in favour of the null hypothesis). The Bayes t-test results were robust to manipulations of the width of the Cauchy prior, this suggests that reported Bayes-factors were not a consequence of a particular choice of statistical prior.

## Discussion

We examined the critical durations for localisation and identification of targets with three types of colouration (edge enhanced disruptive, flat disruptive and uniform) on two types of background; one contrasting with the targets and another matching, the latter affording concealment. Our results show 1) In contrast to our predictions, in the matching background condition localisation critical durations are only longer for edge enhanced targets; 2) in the matching background condition identification critical durations were longer for flat disruptive colouration compared to uniform colouration and longer still for edge enhanced disruptive colouration; 3) There was no difference in localisation critical durations when targets were presented against a contrasting background; 4) When the background contrasted with the targets critical durations are only longer for edge enhanced targets.

Our data illustrate that even when the targets can be reliably located, reliable identification involves a longer presentation time for edge enhanced targets compared to flat disruptive targets or targets with uniform colouration. This dissociation between localisation and identification is crucial for understanding how camouflage works and why animals have evolved their specific colourations. The difference in critical duration may appear relatively small at around 0.2 s (an increase over the uniform control of 37%), however, humans and primates make saccades approximately three times a second^14^ which means that differences of hundredths of second can have a meaningful impact on survival. It is also important to note that all the stimuli were presented very close to fixation on a screen that the participant was observing carefully. In a real-world scenario these effects are likely to be magnified. For example, Egan *et al*.^4^ used a very large search area and recorded search times of several minutes for some edge enhanced targets. The critical durations in the current study ranged from 200–500 ms, this means that the stimuli were detected in three saccades or fewer. One interpretation of the results is that where camouflage is effective, localisation cannot be achieved without confirmatory eye movements towards the target. Reducing the contrast of the background results in faster localisation; no saccade is required. However, in order to correctly *identify* the target the eyes must still move towards the target. These subtle effects would be difficult to measure with a large-scale display because effective camouflage would lead to many more saccades being required before the target was located, creating additional noise that would mask the effect of camouflage patterning. For similar reasons, we also did not consider peripheral viewing, we anticipate that it would be even harder to locate effectively camouflaged targets in the periphery and in many cases identification would be impossible.

The selection pressures driving the evolution of colouration may be more complex than previously imagined, where some patterns of colouration maximize concealment whilst others maximize disguise, the latter may be less tied to a specific habitat. Any distinction depends on the visual characteristics for concealing identity being different from those concealing location. It is well established that flat disruptive camouflage conceals location, through a mechanism of outline disruption^5,6^. However, in contrast to our predictions, we show that flat disruptive camouflage does not conceal identity, in order for this to occur there must be edge enhancement.

The current study illustrates that processes of localisation can be isolated from identification and that some types of camouflage can undermine identification even when they are readily located. However, we cannot conclude that edge enhanced camouflage is qualitatively different from flat disruptive camouflage. The current experiment does not feature a condition in which we have edge-enhancement without disruptive colouration and perforce, the edge-enhanced disruptive stimuli feature larger edge contrasts than the flat disruptive patterns. Therefore, it is not clear whether enhanced edges amplify the known process of outline disruption or take advantage of other visual processes.

Enhanced edges have been shown to facilitate crypsis by giving an impression of pictorial depth^4^. The increased contrast at the edges of coloured patches may exploit processes underlying depth perception. The edges within the animals’ camouflage being perceived as belonging to a nearer object that is occluding the camouflaged object. This could suggest that edge enhanced false edges interfere with the processes underlying surface integration and it is this that is causing the increased identification critical durations. Theories of object recognition differ in their claims about how objects are cortically represented, some propose a 3D volumetric representation^15,16^, while other propose that objects are represented as a collection of visible surfaces^17^. A tantalising interpretation of the current data allied with that of Egan *et al*.^4^ is support for the role of surfaces in object representation, by showing that when camouflage perceptually displaces surfaces, through illusory depth, it significantly slows recognition. Further research is required to establish whether edge-enhancement is specifically suited to undermining object recognition.

## Conclusions

In summary, when background contrast was reduced, hindering concealment, all target types were located equally quickly. However, surprisingly, critical durations for target identification were only longer for edge enhanced disruptive camouflage. This suggests that with some types of disruptive camouflage an animal might be recognised more slowly even when it can be located relatively quickly. The generality of our findings may be wider than anticipated because edge enhancement is a characteristic of many disruptive colouration patterns across many taxa, yet many studies do not include stimuli with enhanced edges. This is also true of artificial camouflage. We suggest that the crypsis of artificial camouflage could be improved by the inclusion of edge enhancement.

## Methods

### Participants

Twenty-seven observers (six male) aged 18–60 participated in the experiment. Two participants were excluded from analysis, leaving a total of twenty-five participants, because they failed to achieve threshold for detection in four or more conditions; i.e. they could not find the targets even at the maximum display duration. We have a relatively small sample size because the power is derived not from testing many participants, but by testing fewer participants many times. All participants had normal or corrected-to-normal vision. Observers gave their informed consent and were treated in accordance with the Declaration of Helsinki (2008, Version 6). Ethical approval for the study was granted by the Abertay University Social and Health Sciences Ethics Panel.

### Stimuli and Apparatus

Stimuli were presented on a gamma-corrected 21-in Sony Trinitron cathode ray tube (CRT) monitor (GDM-F520) with a spatial resolution of 1280 × 1024 pixels and a refresh rate of 60 Hz. All stimuli were presented in the centre of the monitor on a mid-grey background with average luminance of 65.3 cd/m^2^ and with the colour 0.28, 0.30 in CIE xy coordinates – measured with a colourimeter (ColourCal Mk II, Cambridge Research Systems, Cambridge, UK). We used a chin-rest, which ensured a constant viewing distance of 60 cm. All stimuli were generated in Matlab (Version 8.4, The MathWorks Inc., Natick, MA, 2014) and all data were collected using PsychoPy^18^.

The background leaf patterns were generated in the same manner as described in Egan *et al*.^4^ and presented with colours against which targets were relatively hidden (a *matching background*) and with pixel intensities halved (a *contrasting background*) (Fig. 2), stimulus height and width was 28.07° (visual angle). 4096 leaves were placed into each background (mean height = 0.63°, SD = 0.42°). A ‘shadow’ was created beside each leaf by darkening the area immediately to the left of each shape by 30% (in LAB luminance space^19^) the mean width of the shadows was 0.084°.

The target shapes were: wolf, lion, leopard, crocodile, rabbit, gazelle, mouse and stag (see Fig. 1 inset top-right). We were not specifically interested in the true camouflage of these species, the target shapes were merely chosen so that the participants could easily recognise them, the types of camouflage applied to the targets were not intended to reflect any patterning these animals might possess in the real-world. All targets were scaled so that they were composed of roughly the same number of pixels (minimum 22,448 pixels, maximum 22,518 pixels). Mean object height and width were 8.72° (SD = 2.13°) × 6.44° (SD = 2.10°) respectively. Targets were randomly placed within one of four image quadrants (top-left, top-right, bottom-left, bottom-right) by sampling from a uniform distribution of xy coordinates.

There were two types of camouflage (edge enhanced disruptive and flat disruptive), and a uniform coloured control. The patterns were generated in the same manner as described in Egan *et al*.^4^ and had the following properties. Total width of the edge enhancement including both sides of the pattern element boundaries was 0.59° (see inset lower-left Fig. 2). The offset, difference in brightness from the pattern element towards the enhanced edge, was 40 (in CIE L units). The camouflage texture was created using the same means as Egan *et al*.^4^ see Equation 1, except that we used a texture that was spatially slightly coarser (µ = 0.153) in order to match the slightly larger target objects.

The colours composing the leaf and target colouration were selected from a calibrated photograph of a forest floor in Kibale forest (21 for details see 4). The image was converted to CIE LAB space and pixels with values beyond one standard deviation of the a & b axes’ means were excluded. For each camouflaged target stimulus, a light and a dark colour were selected from the retained values based upon the pixel intensity (light: LAB-L values between the 55–65th percentile; dark: LAB-L values between 35–45th percentile). For the uniformly coloured targets, one of either the light or the dark colours was randomly selected. See Supplementary Materials for more stimulus details (Table S1).

### Procedure

Participants were shown a printed sheet with the eight target silhouettes that illustrated which animal fell into each category (predator or prey) and the sheet was available throughout the duration of the experiment. In each trial participants had to correctly identify in which quadrant the target animal was located and whether it was a predator or prey animal. Presentation times were varied using a one-up, three-down staircase procedure. One incorrect response causes presentation times to increase, but three correct responses were required for presentation times to decrease. Thus, causing the staircases to converge at the 79% correct threshold^20^. All staircases started with initial stimulus durations of 2 s. Twelve staircases were randomly interleaved, one for each task, target type and specific target image (2 × 2 × 3). Staircases were terminated after 20 trials. Participants undertook 12 practice trials in order to familiarize themselves with the task. Twenty responses were collected on each staircase for each condition and each task (240 experimental trials per participant). All data and analyses are available on the open science framework^9^.

In a small number of cases the staircases did not converge to a threshold, possibly due to participant inattention or response errors. This was manifested as a larger standard deviation across the final six responses in the staircase. We summed the standard deviation for the 12 conditions that each participant undertook. The mean of these values was 0.0269. The standard deviation of the critical duration of the last six trials of each condition for each participant was calculated, this is equal to 0.032. Those participants whose standard deviations were greater than 1.5 × larger than this, indicating poor performance in the experiment, were excluded from further analysis (two participants). The pattern of statistical results does not change appreciably when the data from these participants are included (these additional analyses are included in the OSF repository see Data Accessibility, below).

### Data accessibility

Data are available on the Open Science Framework osf.io/36dkp.

## Electronic supplementary material

Table S1 and Table S2
