# Supplementary material for: Dissociating the effect of disruptive colouration on localisation and identification of camouflaged targets
Source: Sci Rep. 2018 Apr 26;8:6599. doi: 10.1038/s41598-018-25014-6 (PMC5920097; doi:10.1038/s41598-018-25014-6)
Supplement: Supplementary file 1 — Table S1 and Table S2 [file 41598_2018_25014_MOESM1_ESM.pdf]

Dissociating the effect of disruptive colouration on localisation and identification of camouflaged targets.

Rebecca J. Sharman[1],

Stephen Moncrieff[2],

Paul George Lovell[2].

1. **University of Stirling, School of Psychology, Bridge of Allan, FK9 4LA**
2. **Abertay University, Division of Psychology, School of Social and Health Sciences, 1, Bell St, Dundee, DD1 1HG**

Supplementary Materials.

Table S1: Bayesian ANOVA analysis of results

Model Comparison - dependent

**Models**	**P(M)**	**P(M|data)**	**Log(BF M)**	**Log(BF 10)**	**% error**
Null model (incl. subject)	0.053	8.392e -63	-140.045	0	
camoType	0.053	3.798e -57	-127.022	13.023	0.833
Background Type	0.053	4.670e -53	-117.605	22.44	1.234
camoType + Background Type	0.053	8.864e -46	-100.847	39.199	1.036
camoType + Background Type + camoType ∗ Background Type	0.053	1.073e -43	-96.051	43.995	5.543
task	0.053	5.476e -41	-89.815	50.23	1.986
camoType + task	0.053	2.572e -31	-67.545	72.5	6.419
Background Type + task	0.053	5.185e -25	-53.029	87.017	1.705
camoType + Background Type + task	0.053	1.276e -10	-19.892	120.154	13.962
camoType + Background Type + camoType ∗ Background Type + task	0.053	2.232e -5	-7.82	132.226	5.547
camoType + task + camoType ∗ task	0.053	8.187e -32	-68.69	71.355	1.616
camoType + Background Type + task + camoType ∗ task	0.053	7.539e -11	-20.418	119.627	4.09
camoType + Background Type + camoType ∗ Background Type + task + camoType ∗ task	0.053	2.063e -5	-7.898	132.147	2.266
Background Type + task + Background Type ∗ task	0.053	1.701e -22	-47.235	92.81	3.129
camoType + Background Type + task + Background Type ∗ task	0.053	4.531e -7	-11.717	128.329	3.402
camoType + Background Type + camoType ∗ Background Type + task + Background Type ∗ task	0.053	0.395	2.464	142.007	10.364

camoType + Background Type + task + camoType ∗ task + Background Type ∗ task	0.053	4.365e -7	-11.754	128.291	6.001
camoType + Background Type + camoType ∗ Background Type + task + camoType ∗ task + Background Type ∗ task	0.053	0.51	2.93	142.262	5.004
camoType + Background Type + camoType ∗ Background Type + task + camoType ∗ task + Background Type ∗ task + camoType ∗ Background Type ∗ task	0.053	0.095	0.638	140.583	2.707

Note. All models include subject.

Analysis of Effects - dependent

Effects	P(incl)	P(incl|data)	Log(BF $_{Inclusion}$)
camoType		1	35.707
Background Type		1	35.707
task		1	35.707
camoType ∗ Background Type		1	14.705
camoType ∗ task		0.605	1.2
Background Type ∗ task		1	10.829
camoType ∗ Background Type ∗ task		0.095	0.638

Table S2: Bayesian T-Test Analysis of Results.

		Bayes Paired T-Test		Shapiro-Wilks	
Task		BF_{10}	error %	W	p
**Locate**	**Matching Background**				
	uniform - eeCamo	94715.187	3.704e -11	0.929	0.117
	uniform - flatCamo	0.838	9.273e -5	0.908	0.044
	flatCamo - eeCamo	589.244	1.908e -9	0.958	0.452
	**Contrasting Background**				
	uniform - eeCamo	0.258	3.172e -4	0.878	0.011
	uniform - flatCamo	0.225	3.094e -4	0.911	0.05
	flatCamo - eeCamo	0.249	3.162e -4	0.94	0.194
**Classify**	**Matching Background**				
	uniform - eeCamo	2389.892	6.886e -10	0.949	0.306
	uniform - flatCamo	3.732	1.203e -5	0.974	0.798
	flatCamo - eeCamo	1674.471	4.817e -10	0.979	0.9
	**Contrasting Background**				
	uniform - eeCamo	13.234	6.371e -6	0.962	0.532
	uniform - flatCamo	0.302	3.126e -4	0.926	0.102
	flatCamo - eeCamo	4.756	1.304e -5	0.969	0.688
